# Educational workshops with graduates of the University of Cape Town Karl Storz Head and Neck Surgery Fellowship Program: a model for collaboration in outreach to developing countries

**DOI:** 10.1186/s40064-016-3290-2

**Published:** 2016-09-23

**Authors:** J. J. Fagan, J. Aswani, J. Otiti, V. Mushamba, E. Liyombo, G. Woodson, D. Weed, C. Zender, K. Mannion, J. L. Netterville, R. Wagner, Mark Zafereo

**Affiliations:** 1 Division of Otorhinolaryngology, Faculty of Health Sciences, University of Cape Town, Cape Town, South Africa; 2 Department of Surgery, College of Health Sciences, University of Nairobi, Nairobi, Kenya; 3 Kampala, Uganda; 4 Muhimbili University College of Health Sciences, Muhimbili National Hospital, Dar Es Salaam, Tanzania; 5 Springfield, IL USA; 6 Department of Otolaryngology, University of Miami, Miami, USA; 7 Department of Otolaryngology/ENT Institute, University Hospitals-Case Western Medical Center, Cleveland, OH USA; 8 Department of Head and Neck Surgery, Vanderbilt University Medical Center, Nashville, TN USA; 9 Executive Director Global ENT Outreach, Coupeville, WA USA; 10 Department of Head and Neck Surgery, MD Anderson Cancer Center, 1515 Holcombe Boulevard, Unit 1445, Houston, TX 77030 USA

**Keywords:** Head and neck cancer, Developing countries, Africa, Tanzania, Kenya, Uganda, Rwanda, Surgery, Training, Fellowship, Subspecialty, Outreach, Humanitarian

## Abstract

The University of Cape Town Karl Storz Head and Neck Surgery Fellowship is the only head and neck surgery fellowship in Sub-Saharan Africa. This article briefly describes this fellowship and outlines the experience and ongoing collaborative efforts of members of the American Academy of otolaryngology-head and neck surgery with graduates of this program who are now building head and neck surgery programs in East Africa. This educational collaboration avoids many common pitfalls associated with short-term humanitarian outreach and represents a successful model for international collaborative educational efforts with head and neck surgeons in developing countries in Africa.

## Background

The desire to provide medical care to underserved areas is nothing new to medicine, and represents one of the primary reasons many young women and men continue to choose this vocation. Many physicians in academic and private practices find teaching/training to be one of the most rewarding aspects of their profession. There is a long history of international collaboration in educational efforts and humanitarian activity within otolaryngology-head and neck surgery (Fagan 2012; Isaacson 2014; Pearce et al. 2013). Members of the American Academy of Otolaryngology-Head and Neck Surgery (AAO-HNS) continue to strive to collaborate with international colleagues in supporting educational efforts in developing countries. However, most physicians in both academic and private practices do not have the flexibility to spend extended times abroad in humanitarian activities due to family and work obligations. This article outlines the experience and ongoing collaborative efforts in East Africa with graduates of the University of Cape Town Karl Storz Head and Neck Surgery (UCTKSHNS) Fellowship Program in Cape Town, South Africa and presents a successful model for humanitarian outreach to developing countries.

**Fig. 1:**
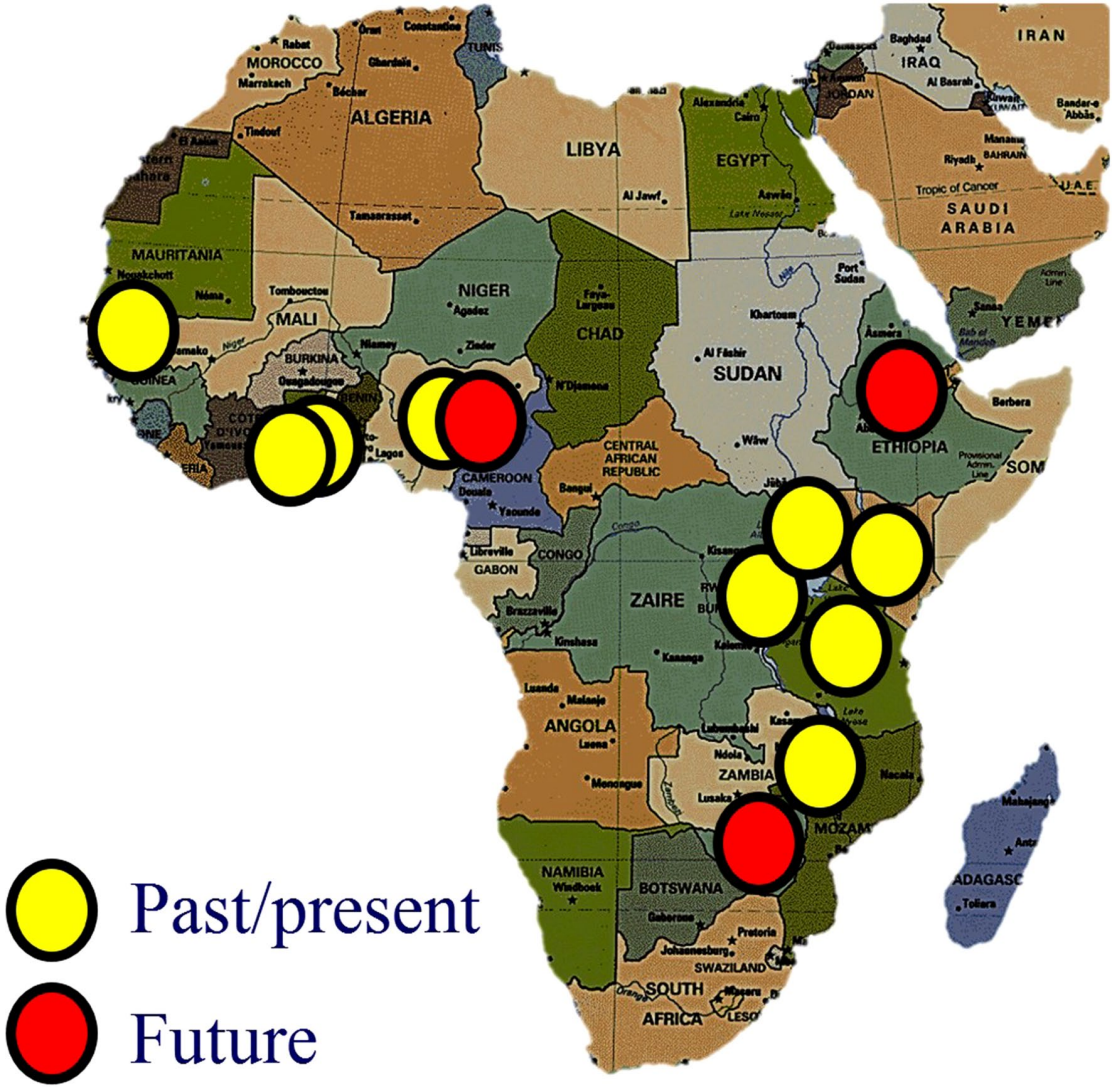
Locations of previous and future head and neck fellows of the University of Cape Town Karl Storz Head and Neck Surgery Fellowship Program

Surgery is the only treatment option for head and neck cancer in many African countries that either do not have radiotherapy facilities, or where facilities are inadequate (Abdel-Wahab et al. 2013). The UCTKSHNS Fellowship is the only head and neck fellowship in Sub-Saharan Africa, and is modelled on similar fellowships in the USA. Fellows are either qualified otolaryngologists or general surgeons. The first 10 fellows have emanated from Uganda, Kenya, Nigeria, Senegal, Ghana (2), Tanzania, Rwanda, Malawi, and currently, Zimbabwe (Fig. 1). All have returned to teaching hospitals in their own countries to establish head and neck programs and teach others what they have learned. The next two fellows are from Nigeria and Ethiopia. Previously there were no trained head and neck surgeons in Sub-Saharan Africa and procedures such as parotidectomy, laryngectomy and neck dissection were not performed or taught in public hospitals in many countries (Fagan and Jacobs 2009).

The University of Cape Town does not have the capacity to provide meaningful ongoing academic support to the fellows upon their return to their home countries. This has created a window of opportunity for members of the AAO-HNS to step in and provide ongoing support for these head and neck surgeons to establish and grow their head and neck programs.

From 2009 to 2014, the authors organized 1–2 week educational workshops with graduates of the UCTKSHNS Fellowship Program. These surgery camps have thus far focused on collaboration with physicians in the East African countries of Kenya, Rwanda, Tanzania, and Uganda. Endeavors were designed to build relationships and to continue to support head and neck surgeons as they finish their head and neck surgery training and transition to academic practice in their respective home countries.

The surgery camps have been organized in major academic centers, often in the National Referral Center hospital in the capital city of the respective country. Hosting these workshops at large hospitals at major teaching institutions has allowed maximal participation of local otolaryngologist-head and neck surgeons, general surgeons, oral surgeons, and residents-in-training. Several of the workshops have been organized to coincide with national ENT society meetings so as to maximize the number of participants. Locating many of the workshops in major teaching hospitals has afforded facilities to incorporate didactic lectures and cadaver labs into the surgery camps; although some workshops have been organized in nearby locations due to operating room constraints at the major university hospitals. Surgical cases following the lectures and cadaver labs have allowed opportunities for patient care and hands-on mutual learning experiences. In many cases, local physicians and residents-in-training have travelled hundreds of miles and across borders to attend these workshops.

Relationships established during these workshops have created further opportunities for educational collaboration for the faculty and residents of the East African programs. Many have thereafter attended AAO-HNS annual meetings and other US otolaryngology-head and neck surgery meetings; as well as participated in short-term visits to US Academic programs.

## Rwanda and Uganda

Dr. Jeffrey Otiti was the 1st graduate of the UCTKSHNS Fellowship in 2006. Two years later, he was invited to set up an ENT department at King Faisal Hospital—the National Referral Hospital in Kigali, Rwanda. In the subsequent years there he initiated a post graduate program in ENT surgery with support from the German ENT Society, who provided visiting ENT specialists for 2 weeks every 4–6 months. The main focus of the ENT support was in General ENT, Otology, and Rhinology.

During his time at King Faisal Hospital, Dr. Otiti co-organized several head and neck surgical workshops with faculty from University of Cape Town and MD Anderson Cancer Center. These courses included didactics, cadaver dissections, and surgeries in the operating theatres; focusing on thyroid surgery, neck dissection, and partial and total laryngeal surgery; with participation of Rwandan faculty and residents in both ENT and general surgery.

In 2013, Dr. Otiti returned to his home country of Uganda to practice at Mulago Hospital/Makere University in Kampala, Uganda. Mulago Hospital is a 1500+ bed hospital, the largest in Uganda, and a National Referral Center for Uganda. Since his return to Uganda, Dr. Otiti has continued to collaborate with AAO-HNS members from Case Western Reserve University and MD Anderson Cancer Center. Faculty from MD Anderson collaborated with Ugandan ENT and general surgery faculty and residents to conduct a thyroid surgery workshop at Mulago Hospital in 2013. Residents from Mbarara, a relatively new and small ENT program 265 km southwest of Kampala, were also able to attend.

One of Dr. Otiti’s achievements has been the initiation of a Head and Neck clinic and Multidisciplinary tumor board at the Uganda Cancer Institute in Kampala. Most recently, through the collaboration of the Ugandan Cancer Institute with Case Western Reserve University, two local and free flap reconstruction workshops were conducted in 2014 and 2015. In addition to other surgeries, several complex free flap reconstruction surgeries were performed.

## Tanzania

Dr. Edwin Liyombo is a senior ENT surgeon consultant/lecturer of otorhinolaryngology at Muhimbili University College of Health Sciences and head of the otorhinolaryngology Department at Muhimbili National Hospital in Dar Es Salaam. Muhimbili National Hospital is a 2000+ bed National Referral Hospital and University Teaching Hospital, the largest and most technologically advanced hospital in the country. Dr. Liyombo has hosted three otolaryngology-head and neck surgery workshops in Tanzania with faculty from MD Anderson Cancer Center. Others including Drs. Gayle Woodson, Tom Robbins, and Richard Wagner have also collaborated with Dr. Liyombo for workshops with his faculty and residents. Workshops have included didactic lectures, cadaver labs, and live surgeries performed collaboratively by Tanzanian and American surgeons, focusing on head and neck diseases covered during didactic sessions and cadaver dissections. Topics of workshops have included thyroid neoplasms, salivary tumors, parapharyngeal space neoplasms, laryngeal tumors, and pectoralis myocutaneous reconstruction of head and neck defects. As Dr. Liyombo is the current president of the Tanzanian Otorhinolaryngology Society, the workshop in 2013 was coordinated with the annual Tanzanian Otorhinolaryngology Society meeting, such that approximately 90 % of the otorhinolaryngologists and residents in training in the country were able to participate.

The workshops have typically been organized with 1–2 days of didactics, 1–2 days of cadaver dissection, and 2–3 days of hands-on surgery for patients who have been selected over the preceding months by the faculty and residents at Muhimbili National Hospital. Organizing the workshops at Muhimbili National Hospital has ensured that patients have appropriate care and follow-up with faculty and residents following the surgery camps. The number of days in the operating room has typically been limited to 2–3 days so as not to overwhelm the resources of the hospital and staff.

Relationships established during these workshops have provided opportunities for faculty at Muhimbili to visit academic centers in the United States and attend AAO-HNS annual meetings. Additionally, these workshops have provided inspiration for one of the young faculty at Muhimbili to pursue additional head and neck surgery training. Dr. Victor Mashamba, who has participated in these workshops as both resident and faculty, recently completed the UCTKSHNS fellowship and has returned to Muhimbili to help establish a head and neck surgery program. Ongoing collaborative efforts continue, with plans for another head and neck surgery workshop in conjunction with the 2015 Tanzanian ENT Society meeting in Moshi.

## Kenya

Kenya has held two head and neck workshops and two surgical camps following the return of Dr. Joyce Aswani, also a UCTKSHNS fellow. The first workshop was held in October 2009 in Nairobi and included the ENT-HNS, maxillofacial, and plastic and reconstructive teams, who collectively form the backbone of the Multidisciplinary Head and Neck Surgery team at Kenyatta National Hospital. The faculty consisted of a two surgeons from South Africa, a head and neck surgeon from Cape Town and a plastic and reconstruction surgeon from Pretoria. In attendance were surgeons and residents from Uganda, Burundi and Kenya. The course covered a day of lectures, a day of cadaver dissection, and a day of live surgeries transmitted to the lecture theatres by video teleconferencing. The second workshop was held in August 2013 in Nairobi. Participants were from Rwanda, Uganda, Zambia and Kenya. The faculty consisted of AAO-HNS members from Vanderbilt and Harvard Universities. The course content again consisted of lectures, cadaver dissection, and live surgeries over a period of 3 days. A pre- and post-course self-evaluation survey showed an increase in the number of participants able to complete the specific surgeries (Chambers et al. 2015).

In 2014, two 2-week head and neck surgical camps were held in Malindi, Kenya with over 100 patients undergoing surgery. The focus of the camps has been to transfer skills and medical equipment from AAO-HNS faculty to local ENT physicians and residents. At least 15 Kenyan surgeons with an interest in head and neck surgery have participated, representing about 25 % of all otolaryngologists in Kenya. Additionally, clinical officers from Kenyatta National Hospital received training in post-laryngectomy speech rehabilitation. Since operating room time at national teaching hospitals in Kenya can be extremely limited, a unique aspect of these camps was the use of operating rooms in collaboration with a private hospital in Malindi, which afforded facilities to accommodate a large volume of patients over a short time period. This hybrid environment of a collaborative training workshop in a private setting but coordinated by faculty from Kenyatta National Hospital allowed maximal patient care and educational opportunities during the 2 weeks, while also ensuring appropriate local follow-up with Kenyatta surgeons.

## Conclusions

Although short 1–2 week collaborative educational workshops provide invaluable opportunities to build relationships with international colleagues, satisfy an inherent desire to help others and share knowledge, and provide exciting opportunities to learn and experience other cultures, such short medical humanitarian trips can also present dilemmas. These include uncertain patient selection and follow-up, potential disruption to local medical communities, and language/cultural gaps (Welling et al. 2010). Visiting doctors also must take great care not to undermine the standing and authority of established local medical colleagues.

This article has outlined examples of how targeted 1–2 week outreaches by AAO-HNS otolaryngologists-head and neck surgeons have supplemented training in head and neck surgery on the African continent through building relationships with and supporting colleagues who have completed UCTKSHNS Fellowships. These workshops have provided rewarding international collaboration while seeking to avoid some common pitfalls associated with short-term humanitarian medical efforts. The most important thing that the authors have learned from their early experience is that the success of each individual workshop has largely depended upon the leadership of the UCTKSHNS Fellowship graduate, who has served as a point person in each respective country to best organize and optimize patient and physician participation. The leadership of the UCTKSHNS Fellowship graduates in facilitating these workshops specifically served to provide continuity of care for patients, create enthusiasm and increased participation from the local medical community, and bridge cultural and language gaps between visiting surgeons and patients on the African continent. Focus of future efforts should include more formal outcome measurements to guide continued improvement and modifications in patient care and educational endeavors. Organizing groups of AAO-HNS members who can provide coordinated ongoing support to international otolaryngology-head and neck surgery colleagues in developing countries remains a challenge, but provides a great opportunity to advance the teaching and practice of head and neck surgery in developing countries.
